# Supplementary material for: CENP-A and CENP-B collaborate to create an open centromeric chromatin state
Source: Nat Commun. 2023 Dec 12;14:8227. doi: 10.1038/s41467-023-43739-5 (PMC10716449; doi:10.1038/s41467-023-43739-5)
Supplement: Supplementary file 1 — Supplementary Information [file 41467_2023_43739_MOESM1_ESM.pdf]

Supplementary Tables

Supplementary Table 1

Fragment	Sequence
1x601	-100 -95 -90 -85 -80 -75 -70 -65 -60 -55 -50 -45 -40 -35 -30 -25<br>GATCGGTCTCATAGC**CTGGAGAATCCCGGTGCCGAGGCCGCTCAATTGGTCGTAGACAGCTCTA**<br>-20 -15 -10 -5 0 5 10 15 20 25 30 35 40 45 50 55<br>**GCACCGCTTAAACGCACGTACGCGCTGTCCCCCGCGTTTTAACCGCCAAGGGGATTACTCCCTAGTCTCCAGGCACGTGT**<br>60 65 70 75 80 85 90 95 100 105<br>**CAGATATATACATCCTGTCACACTGTGGATC**
1x601 BB	-100 -95 -90 -85 -80 -75 -70 -65 -60 -55 -50 -45 -40 -35 -30 -25<br>GATCGGTCTCATAGCCTTCGTTGGAAACGGGA**GAATCCCGGTGCCGAGGCCGCTCAATTGGTCGTAGACAGCTCTA**<br>-20 -15 -10 -5 0 5 10 15 20 25 30 35 40 45 50 55<br>**GCACCGCTTAAACGCACGTACGCGCTGTCCCCCGCGTTTTAACCGCCAAGGGGATTACTCCCTAGTCTCCAGGCACGTGT**<br>60 65 70 75 80 85 90 95 100 105<br>**CAGATATATACATCCTGT**CACACTGTGGATC
P1 DA1 BB	-100 -95 -90 -85 -80 -75 -70 -65 -60 -55 -50 -45 -40 -35 -30 -25<br>CACTTGGTGGCGGCCGCC**CTGGAGAATCCCGGTGCCGAGGCCGCTCAATTGGTCGTAGACAGCTCTA**<br>-20 -15 -10 -5 0 5 10 15 20 25 30 35 40 45 50 55<br>**GCACCGCTTAAACGCACGTACGCGCTGTCCCCCGCGTTTTAACCGCCAAGGGGATTACTCCCTAGTCTCCAGGCACGTGT**<br>60 65 70 75 80 85 90 95 100 105<br>**CAGATATATACAAGATCT**AGTACTTGGTCTCATAGC
P1 DA3 BB	-100 -95 -90 -85 -80 -75 -70 -65 -60 -55 -50 -45 -40 -35 -30 -25<br>CACTTGGTGGCGGCCGCC**CTGGAGAATCCCGGTGCCGAGGCCGCTCAATTGGTCGTAGACAGCTCTA**<br>-20 -15 -10 -5 0 5 10 15 20 25 30 35 40 45 50 55<br>**GCACCGCTTAAACGCACGTACGCGCTGTCCCCCGCGTTTTAACCGCCAAGGGGATTACTCCCTAGTCTCCAGGCACGTGT**<br>60 65 70 75 80 85 90 95 100 105<br>**CAGATATATACAAGATCT**TCTCTAGATCCATGGAGTACTTGGTCTCATAGC
P2 DA1 BB	-100 -95 -90 -85 -80 -75 -70 -65 -60 -55 -50 -45 -40 -35 -30 -25<br>CTTCGTTGGAAACGGGA**GAATCCCGGTGCCGAGGCCGCTCAATTGGTCGTAGACAGCTCTA**<br>-20 -15 -10 -5 0 5 10 15 20 25 30 35 40 45 50 55<br>**GCACCGCTTAAACGCACGTACGCGCTGTCCCCCGCGTTTTAACCGCCACGAGGATTACTCCCTAGTCTCCAGGCACGAGC**<br>60 65 70 75 80 85 90 95 100 105<br>**CAGATATATACATCCTGTCACACTGTGCCAA**
P2 DA3 BB	-100 -95 -90 -85 -80 -75 -70 -65 -60 -55 -50 -45 -40 -35 -30 -25<br>C**TGGAGAATCCCGGTGCCGAGGCCGCTCAATTGGTCGTAGACAGCTCTA**<br>-20 -15 -10 -5 0 5 10 15 20 25 30 35 40 45 50 55<br>**GCACCGCTTAAACGCACGTACGCGCTGTCCCCCGCGTTTTAACCGCCAAGGGGATTACTCCCTAGTCTCCAGGCTCGTGT**<br>60 65 70 75 80 85 90 95 100 105<br>**CAGATATATACATCCTGT**CACACTGTGCCAAGTACT
P3 DA1 BB	-100 -95 -90 -85 -80 -75 -70 -65 -60 -55 -50 -45 -40 -35 -30 -25<br>GTACTTACGCGGCCGCCC**TGGAGAATCCCGGTGCCGAGGCCGCTCAATTGGTCGTAGACAGCTCTA**<br>-20 -15 -10 -5 0 5 10 15 20 25 30 35 40 45 50 55<br>**GCACCGCTTAAACGCACGTACGCGCTGTCCCCCGCGTTTTAACCGCCAAGGGGATTACTCCCTAGTCTCCAGGCACGTGT**<br>60 65 70 75 80 85 90 95 100 105<br>**CAGATACTGCAGAGATCT**AGTACTTGGTCTCAAACC
P3 DA3 BB	-100 -95 -90 -85 -80 -75 -70 -65 -60 -55 -50 -45 -40 -35 -30 -25<br>CTTCGTTGGAAACGGGA**GAATCTCGTGGCCGAGGCCGCTCAATTGGTCGTAGACAGCTCTA**<br>-20 -15 -10 -5 0 5 10 15 20 25 30 35 40 45 50 55<br>**GCACCGCTTAAACGCACGTACGCGCTGTCCCCCGCGTTTTAACCGCCAAGGGGATTACTCCCTAGTCTCCAGGCACGTGT**<br>60 65 70 75 80 85 90 95 100 105<br>**CAGATACTGCAGAGATCT**CACGAGCCATGGAGTACTTGGTCTCAAACC
P4 DA1 BB	-100 -95 -90 -85 -80 -75 -70 -65 -60 -55 -50 -45 -40 -35 -30 -25<br>CTTCGTTGGAAACGGGA**GAATCCCGGTGCCGAGTCCGCTCAATTGGTCGTAGAGTCCTCTA**<br>-20 -15 -10 -5 0 5 10 15 20 25 30 35 40 45 50 55<br>**GCACCGCTTAAACGCACGTACGCGCTGTCCCCCGCGTTTTAACCGCCAAGGGGATTACTCCCTAGTCTCCAGGCACGTGT**<br>60 65 70 75 80 85 90 95 100 105<br>**CAGATATATACATCC**TGTCACGTCGTGCCAA

P4 DA3 BB	-100 -95 -90 -85 -80 -75 -70 -65 -60 -55 -50 -45 -40 -35 -30 -25<br>**CACGAGAATCCCGGTGCCGAGGCCGCTCAATTGGTCGTAGACAGCTCTA**<br>-20 -15 -10 -5 0 5 10 15 20 25 30 35 40 45 50 55<br>**GCACCGCTTAAACGCACGTACGCGCTGTCCCCCGCGTTTTAACCGCCAAGGGGATTACTCCCTAGTCTCCAGGCACGTGT**<br>60 65 70 75 80 85 90 95 100 105<br>**CAGATATATACATCC**TGTCACGTCGTGCCAA
P5	-100 -95 -90 -85 -80 -75 -70 -65 -60 -55 -50 -45 -40 -35 -30 -25<br>GTACTTACGCGGCCGCC**CTGGAGAATCCCGGTGCCGAGGCCGCTCAATTGGTCGTAGACAGCTCT**<br>-20 -15 -10 -5 0 5 10 15 20 25 30 35 40 45 50 55<br>**AGCACCGCTTAAACGCACGTACGCGCTGTCCCCCGCGTTTTAACCGCCAAGGGGATTACTCCCTAGTCTCCAGGCACGTG**<br>60 65 70 75 80 85 90 95 100 105<br>**TCAGATACTGCAGAGATCT**CTAGATCCGGTCTCACTAA
α-satel-lite (171)	-100 -95 -90 -85 -80 -75 -70 -65 -60 -55 -50 -45 -40 -35 -30 -25<br>GGAGGATTTCGTTGGAAACGGGATCAACTTCCCATAACTGAACGGAAGCAAACTCAGAACATTC<br>-20 -15 -10 -5 0 5 10 15 20 25 30 35 40 45 50 55<br>TTTGTGATGTTTGTATTCAACTCACAGAGTTGAACCTTCCTTTGATAGTTCAGGTTTGCAACACCCTTGTAGTAGAATCT<br>60 65 70 75 80 85 90 95 100 105<br>GCAAGTGTATATTTTGACCACTTTGGA

Supplementary Table 1 | Sequences of 1 x 601 pieces for recombinant and PCR-generated DNA pieces. 601 sequences indicated in bold. The labeled base pairs are indicated in red. The numbering is given as number of base-pairs relative to the dyad in the 601 sequence. B-Box (CENP-B binding) sites are indicated in blue. From these sequences, chromatin fiber DNA is assembled as described in Supplementary Table 3.

Supplementary Table 2

Description	Dye	Sequence
P2_anchor_pos39_rev	Alexa 647	5'-biotin-GATCCACAGTGTGACAGGATGTATATATCTGACACGTGCC TGGAGAC/iAmMC6T/AGGGAG-3'
P5_PNP_anchor_antisense_dye	Alexa 647	5'-ph-AGCTTAGTCTGC/iAmMC6T/CAGTACTCGTCGCTAGATCCATG GTCCGATTACGCGG-3'
P5_PNP_anchor_antisense	-	5'-ph-AGCTTAGTCTGCTCAGTACTCGTCGCTAGATCCATGGTCCGATT ACGCGG-3'
P5_PNP_anchor_sens	-	5'-biotin-CCGCGTAATCGGACCATGGATCTAGCGACGAGTACTGAGC AGACT-3'
P3_DA3BB_pos82_PNP_insert	Alexa 647	5'-ph-TCGTGGGTTTGAGACCAAGTACTCCA/iAmMC6T/GGC-3'
P2_DA3BB_pos88_PNP_insert	Cy3B	5'-ph-TCGTGTCAGATATATACATCCTGTCACACTGTGCCAAG/iAmMC6T/ ACTCTTCGT TGGAAACGGAAGAATC-3'
P2_DA1_pos39_PNP_insert	Cy3B	5'-ph-TCGTGCCTGGAGAC/iAmMC6T/AGGGAGTAATCC-3'
P4_DA1_pos-39_PNP_insert	Alexa 647	5'-Ph-CAATTGG/iAmMC6T/CGTAGAGTCCTCT-3'
α-satellite forward	Cy3	Cy3-GGAGGATTTCGTTGGAAACG-3'
α-satellite reverse	Cy5	Cy5-TCCAAAGTGGTCAAAATATACACTTG-3'

Supplementary Table 2 | Sequences of all labeled oligonucleotides. Ph: 5' phosphorylation. iAmMC6T: internal amino-modified C6 dT linker, used for dye attachment.

Supplementary Table 3

Experiment	Name	Backbone	Dye	Modification
CENP-B binding	1x601	P3_DA1BB	Alexa647 (39)	3' biotin
	1x601 BB	P3_DA3BB	Alexa647 (39)	3' biotin
CENP-B chromatin binding	DA3BB	P1,P2;P3DA3BB;P4,P5	P5_anchor: Alexa647	3' biotin
Chromatin FRET	CH DA1BB FRET	P1 ; P2DA1BB ; P3 ; P4DA1BB ; P5	P2: Cy3B (39), P4: Alexa647 (-39)	3' biotin
	CH DA3BB FRET	P1 ; P2 ; P3DA3BB ; P4 ; P5	P2: Cy3B (88), P3: Alexa647 (82)	3' biotin
Nucleosome FRET	α-sat_Cy3_Cy5	α-satellite	Cy3, Cy5	N/A

Supplementary Table 3 | Overview of all chromatin DNA with different combinations of labels. The DNA is composed of sequence elements in the order as described under 'Backbone', using sequences from **Supplementary Table 1**. For chromatin DNA, using the plug-and-play strategy (**Supplementary Fig. 1**), indicated labeled sequences (see **Supplementary Table 2**) are swapped in and ligated.

Supplementary Table 4

		**CENP-A DA1BB FRET**		**CENP-A DA3BB FRET**		**H3 DA1BB FRET**		**H3 DA3BB FRET**	
		**0 mM Mg^{2+}**	**4 mM Mg^{2+}**	**0 mM Mg^{2+}**	**4 mM Mg^{2+}**	**0 mM Mg^{2+}**	**4 mM Mg^{2+}**	**0 mM Mg^{2+}**	**4 mM Mg^{2+}**
**LF**	A1	5.51 ± 0.8	4.9 ± 0.8	6.81 ± 0.42	3.72 ± 0.78	2.24 ± 1.18	2.52 ± 0.44	2.27 ± 0.4	x
	c1	0.036 ± 0.022	0.056 ± 0.03	0.07 ± 0.02	0.074 ± 0.006	0.047 ± 0.035	0.05 ± 0.02	0.10 ± 0.03	x
	s1	0.1 ± 0.03	0.1 ± 0.02	0.05 ± 0.004	0.06 ± 0.008	0.06 ± 0.03	0.12 ± 0.03	0.084 ± 0.022	x
	% area	77.74 ± 13.8	61.76 ± 6.46	39.78 ± 6.62	29.4 ± 9.68	19.67 ± 12.72	38.37 ± 2.18	16.03 ± 13.93	x
**HF**	A2	1.28 ± 0.73	2.13 ± 0.3	3.43 ± 0.36	4.47 ± 0.67	6.57 ± 1.04	3.96 ± 0.59	7.04 ± 1.04	6.81 ± 0.7
	c2	0.19 ± 0.05	0.44 ± 0.05	0.18 ± 0.02	0.32 ± 0.002	0.26 ± 0.036	0.57 ± 0.01	0.36 ± 0.035	0.47 ± 0.02
	s2	0.12 ± 0.05	0.14 ± 0.03	0.13 ± 0.002	0.012 ± 0.004	0.09 ± 0.015	0.125 ± 0.016	0.09 ± 0.026	0.10 ± 0.016
	% area	22.25 ± 13.8	38.2 ± 6.46	60.21 ± 6.62	70.6 ± 9.68	80.32 ± 12.72	61.6 ± 2.18	83.96 ± 13.93	100
**N total traces**		404	483	366	335	404	530	417	418
**N dynamic traces**		x	129	x	53	x	32	x	15
**% dynamic traces**		x	25.24 ± 4.72	x	15.6 ± 0.64	x	6.0 ± 2.3	x	2.9 ± 2.3
**N of repeats**		4	4	2	2	4	3	3	3

Supplementary Table 4 | Summary of Gaussian fits and percentage of dynamic traces from chromatin compaction experiments. Errors are fitting errors. N dynamic traces are determined by the existence of anticorrelated intensity fluctuations in donor- and acceptor channels.

Supplementary Table 5

	dissociation kinetics				binding kinetics		
	dwell time (s)		Amplitude (%)		rate constants (x 10^7 M*s-1)		
	$\tau_{off,1}$	$\tau_{off,2}$	A_0	A_1	k_{on}	$k_{on, per\ DNA\ repeat}$	n exp.
**DNA**							
1x 601	1.68 ± 0.5	20.89 ± 5.8	65 ± 8	34 ± 8	8.1 ± 4.6	8.1 ± 4.7	4
1x601 BB	6.94 ± 1.42	61.76 ± 11.8	49 ± 9.5	51 ± 9.5	9.9 ± 2.5	9.9 ± 2.5	4
**Nucleosomes**							
H3 1x601 MN	3.4 ± 0.8	15.2 ± 1	51 ± 3.5	49 ± 3.5	14.4 ± 1.18	14.4 ± 1.18	2
H3 1x601 BB MN	6.87 ± 2	48.3 ± 3.9	66 ± 1.6	34 ± 1.6	9.6 ± 0.4	9.6 ± 0.4	2
CA 1x601 BB	6.8 ± 2	50.21 ± 6.8	51 ± 10	49 ± 10	6.6 ± 1.6	6.6 ± 1.6	2
**Chromatin**							
H3 DA3 (1xBB)	3.6 ± 2.0	17.6 ± 6.7	47 ± 22	52 ± 22	5.7 ± 1.3	0.48 ± 0.11	3
CA DA3 (1xBB)	4.9 ± 0.46	51.39 ± 5.3	67 ± 2.7	33 ± 2.7	14 ± 3.2	1.2 ± 0.27	4
**DNA -CENP-B 1-150**							
1x 601	0.9 ± 0.06	5.2 ± 0.9	59 ± 4	41 ± 4	9.2 ± 1.8	9.2 ± 1.8	4
1x601 BB	2.6 ± 0.15	22.82 ± 4.4	71 ± 0.03	29 ± 0.03	11.1 ± 3.1	11.1 ± 3.1	4

Supplementary Table 5 | All kinetic parameters of CENP-B interacting with DNA, nucleosomes and chromatin. Under the measurement conditions, fluorophore lifetime was determined to be 168 s (see ref. [1]). Reported rate constants are not further corrected for photobleaching.

Supplementary Table 6

**Data collection**	**H3 $^{\alpha\text{-sat}}$ nucleosome**	**CENP-A$^{\alpha\text{-sat}}$ nucleosome**	
Magnification	105 000x	105 000x	
Voltage (kV)	300	300	
Electron exposure (e–/Å^2)	57.5	57.5	
Defocus range (µm)	-0.6 – -2.2	- 0.2 – -2.2	
Pixel size (Å)	0.85	0.85	
**H3 $^{\alpha\text{-sat}}$ nucleosome data processing**	**Class 1**<br>EMD- 18699<br>(Most wrapped)	**Class 2**<br>EMD- 18714<br>(Most unwrapped)	
Initial reference map	ab initio	ab initio	
Symmetry imposed	C1	C1	
Initial particle images (no.)	326 847	326 847	
Final particle images (no.)	242 755	84 092	
Box size (Å)	160	160	
Pixel size (Å)	1.58	1.58	
Map resolution (Å)<br>FSC threshold	3.2	3.7	
**CENP-A$^{\alpha\text{-sat}}$ nucleosome data processing**	**Class 1**<br>EMD- 18739<br>(Most wrapped)	**Class 3**<br>EMD- 18740<br>(Partially unwrapped)	**Class 5**<br>EMD- 18745<br>(Most unwrapped)
Initial reference map	ab initio	ab initio	ab initio
Symmetry imposed	C1	C1	C1
Initial particle images (no.)	926 948	926 948	926 948
Final particle images (no.)	250 755	179 623	87 222
Box size (Å)	160	160	160
Pixel size (Å)	1.58	1.58	1.58
Map resolution (Å), FSC threshold	3.93	3.93	4.66

Supplementary Table 6 | Cryo-EM data collection and map refinement statistics for H3$^{\alpha\text{-sat}}$ nucleosome and CENP-A$^{\alpha\text{-sat}}$ nucleosome.

Supplementary Table 7

	Class 1<br>EMD- 18753<br>(Most wrapped)	Class 2<br>EMD- 18763<br>(Partially unwrapped)	Class 3<br>EMD- 18768<br>(Most unwrapped)
**Data collection**			
Magnification	165 000x	165 000x	165 000x
Voltage (kV)	300	300	300
Electron exposure (e–/Å^2)	50	50	50
Defocus range (μm)	-1.2 – -2.4	-1.2 – -2.4	-1.2 – -2.4
Pixel size (Å)	0.704	0.704	0.704
**Data processing**			
Initial reference map	ab initio	ab initio	ab initio
Symmetry imposed	C1	C1	C1
Initial particle images (no.)	277 488	277 488	277 488
Final particle images (no.)	35 239	101 005	141 244
Box size (Å)	160	160	160
Pixel size (Å)	1.58	1.58	1.58
Map resolution (Å), threshold	4.06	4.05	3.89

Supplementary Table 7 | Cryo-EM data collection and map refinement statistics for CENP-A$^{\alpha\text{-sat}}$ nucleosome + CENP-B.

Supplementary Table 8

	Class 3<br>EMD- 18775<br>(free CENP-A$^{601\ BB}$ nucleosome)	Class 5<br>EMD-18776<br>(bound CENP-A$^{601\ BB}$ nucleosome)
**Data collection**		
Magnification	105 000x	105 000x
Voltage (kV)	300	300
Electron exposure (e–/Å^2)	57.5	57.5
Defocus range (μm)	-0.6 – -2.2	-0.6 – -2.2
Pixel size (Å)	0.83	0.83
**Data processing**		
Initial reference map	ab initio	ab initio
Symmetry imposed	C1	C1
Initial particle images (no.)	878 365	878 365
Final particle images (no.)	246 772	38 854
Box size (Å)	160	360
Pixel size (Å)	1.86	0.83
Map resolution (Å), FSC threshold	3.81	3.1

Supplementary Table 8 | Cryo-EM data collection and map refinement statistics for CENP-A$^{601\ BB}$ nucleosome + CENP-B.

Supplementary Table 9

		CENP-A DA1BB FRET			CENP-A DA3BB FRET		H3 DA1BB FRET		H3 DA3BB FRET	
**CENP-B conc.**		**500 pM**	**5 nM**	**50 nM**	**5 nM**	**50 nM**	**5 nM**	**50 nM**	**5 nM**	**50 nM**
**LF**	**A1**	5.5 ± 0.26	5.98 ± 0.79	4.55 ± 2.16	5.5 ± 0.05	5.11 ± 0.56	2.74 ± 0.53	3.92 ± 0.12	1.72 ± 0.03	1.54 ± 0.89
	**c1**	0.02 ± 0.02	0.008 ± 0.002	0.04 ± 0.05	0.08 ± 0.04	0.06 ± 0.05	0.05 ± 0.04	0.05 ± 0.002	0.11 ± 0.01	0.08 ± 0.01
	**s1**	0.08 ± 0.004	0.08 ± 0.014	0.11 ± 0.05	0.08 ± 0.01	0.074 ± 0.008	0.14 ± 0.04	0.12 ± 0.001	0.118 ± 0.002	0.1 ± 0.02
	**% area**	56.9 ± 6.3	65.87 ± 2.02	68.2 ± 4.7	57.9 ± 8.61	49.17 ± 0.19	46.07 ± 3.4	59.64 ± 2.12	25.64 ± 1.49	19.4 ± 7.4
**HF**	**A2**	1.95 ± 0.18	1.65 ± 0.04	1.19 ± 0.35	2.93 ± 0.09	3.73 ± 0.09	3.24 ± 0.17	2.30 ± 0.4	6.62 ± 0.36	6.66 ± 0.21
	**c2**	0.35 ± 0.01	0.42 ± 0.04	0.41 ± 0.2	0.31 ± 0.03	0.24 ± 0.06	0.54 ± 0.05	0.53 ± 0.015	0.46 ± 0.04	0.443 ± 0.008
	**s2**	0.17 ± 0.044	0.16 ± 0.003	0.20 ± 0.01	0.11 ± 0.027	0.10 ± 0.002	0.13 ± 0.013	0.14 ± 0.03	0.09 ± 0.008	0.096 ± 0.003
	**% area**	43.1 ± 6.3	34.12 ± 2.02	31.8 ± 4.7	42.09 ± 8.61	50.82 ± 0.19	53.92 ± 2.02	40.35 ± 2.12	74.35 ± 1.49	80.55 ± 7.4
**N total traces**		206	181	189	354	304	663	389	289	248
**N dynamic traces**		35	34	16	121	43	64	37	18	17
**% dynamic traces**		16.5 ± 6	18.0 ± 3.8	7.6 ± 3.1	34.6 ± 1.4	15.7 ± 4.8	9.9 ± 1.7	9.6 ± 0.7	6.1 ± 1.5	6.8 ± 5.1
**N of repeats**		2	2	2	2	2	3	2	2	2

Supplementary Table 9 | Summary of Gaussian fits from chromatin remodeling induced by CENP-B invasion experiments.

Supplementary Figures

Supplementary Fig. 1

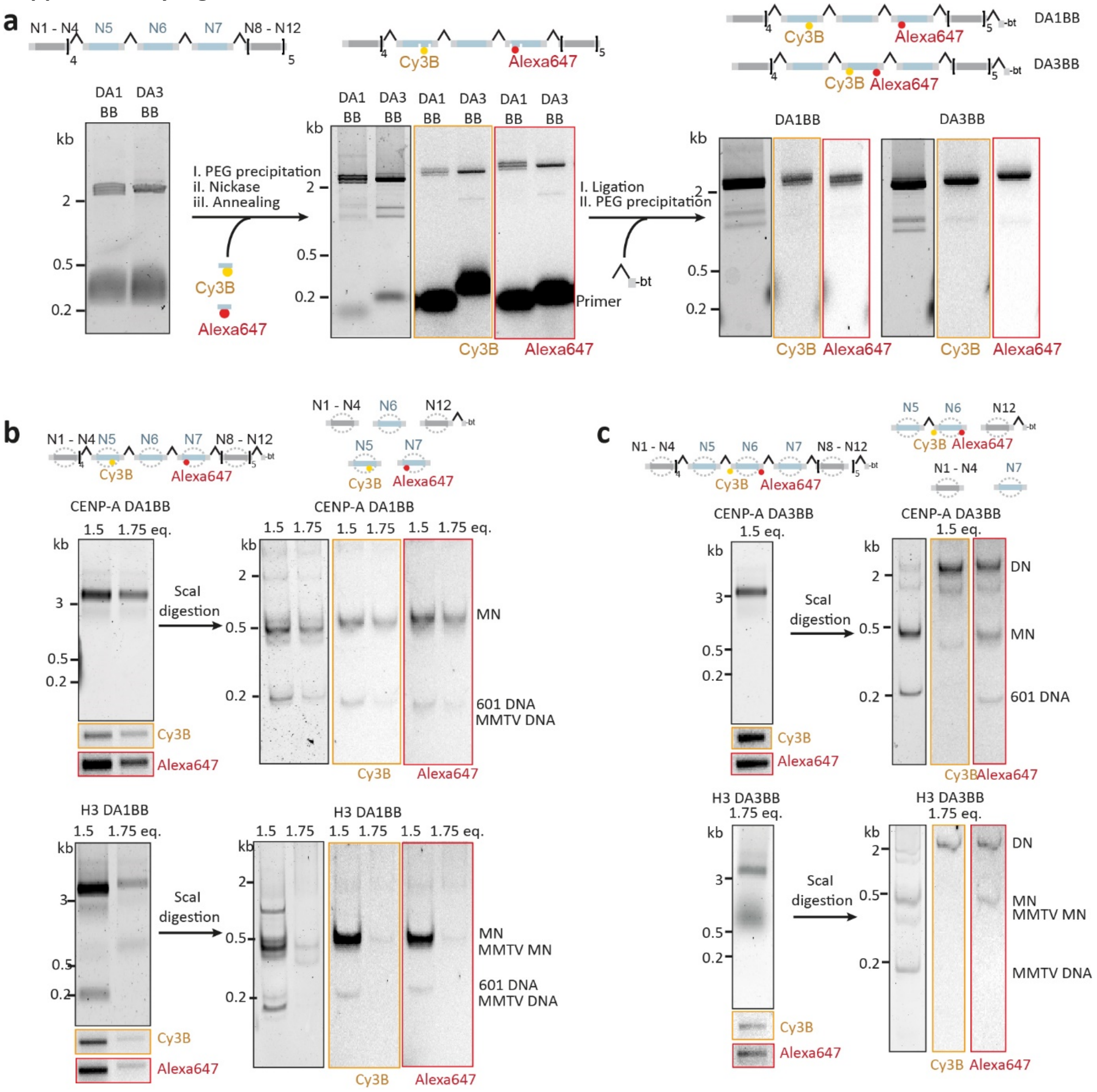

Supplementary Fig. 1 - Chromatin assembly. a Scheme and native PAGE analysis of DNA assembly containing FRET donor (Cy3B, yellow) and acceptor (Alexa fluor 647, red) at nucleosome N5 and N7 (DA1) and at N6 (DA3) on B-Box containing (BB) DNA using plug-and-play DNA ligations[2]. 12 x 601 NPS containing DNA is purified and nicked by nicking endonucleases to create gaps into which fluorophore containing primers are annealed. All gels are imaged using GelRed, and fluorescence imaging for Cy3B and Alexa647. For DNA constructs, see Supplementary Tables 1-3. **b** Preparation of CENP-A (top) and H3 (bottom) containing chromatin arrays on DA1BB DNA template. Saturated arrays are shown at 1:1.75 DNA to histone octamer ratio for CENP-A and 1:1.5 DNA to octamer ration for H3. Saturation is confirmed by ScaI digestion and SDS PAGE analysis of the digestion products. MMTV DNA is used for H3 as a low affinity histone buffer. For DNA constructs, see Supplementary Tables 1-3. **c** Preparation of CENP-A (top) and H3 (bottom) containing DA3BB chromatin arrays. Saturated arrays are shown at 1:1.5 DNA to octamer ratio for CENP-A and 1:1.75 DNA to octamer ration for H3. Saturation is confirmed by ScaI digestion. As the ScaI site is obstructed by the cy3B fluorophore we see most labelled nucleosomes as dinucleosomes. For DNA constructs, see Supplementary Tables 1-3

Supplementary Fig. 2

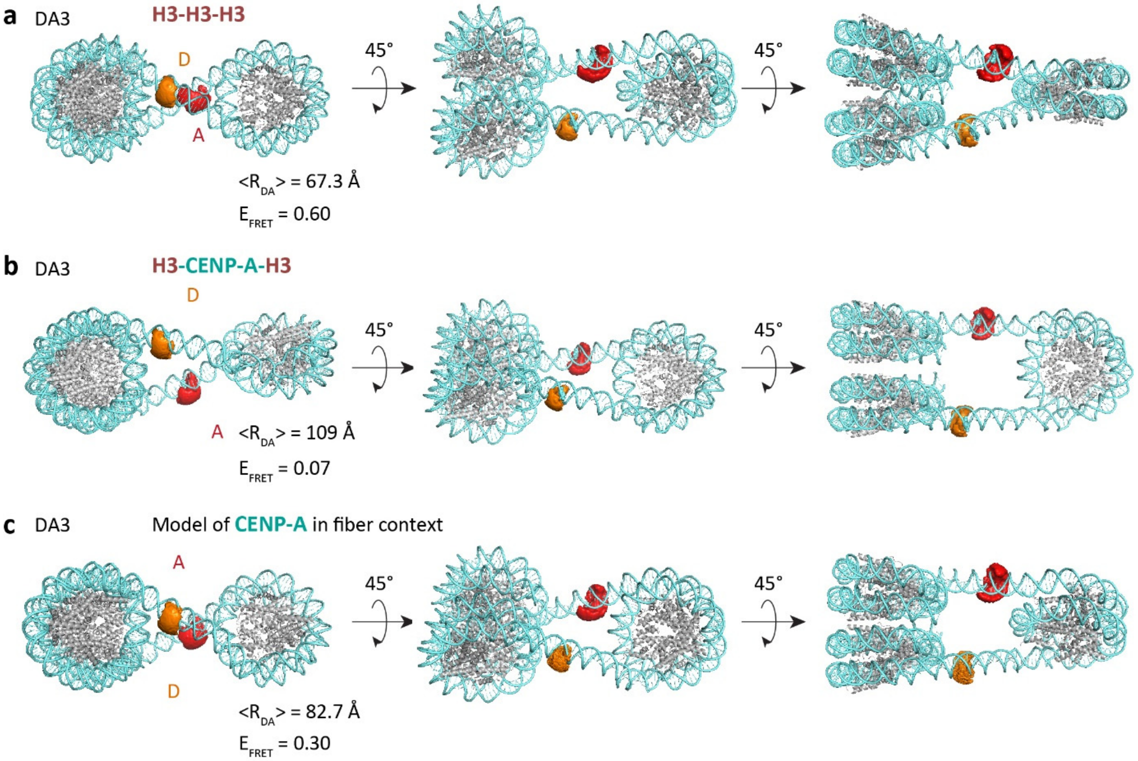

Supplementary Fig. 2 – Calculated inter-dye distances and E_{FRET} values for DA3 H3 and CENP-A chromatin structures. a H3-H3-H3 trinucleosome structure from ref. [3], PDB code 6l4a, with DA3 FRET pair, in the DNA ‘outward path’ orientation. Inter-dye distances based on dye accessible volumes (AV) were calculated using the FRET positioning and screening toolkit[4]. Calculated E_{FRET} values (E_{FRET} = 0.6) are comparable to experimentally observed values for H3 (E_{FRET} = 0.48) chromatin fibers (see Fig. 2). **b** H3-CENP-A-H3 trinucleosome structure from ref. [3], PDB code 6l49. The calculated inter-dye distance $\langle R_{DA} \rangle$ = 109 Å is too large to obtain measurable FRET in this conformation. **c** Chromatin state compatible with observed FRET values. Here, the conformation lies in between a H3-H3-H3 and H3-CENP-A-H3 array, with an intermediate twist of the central nucleosome, resulting in a calculated E_{FRET} value of 0.3, close to the observed value of E_{FRET} values = 0.32.

Supplementary Fig. 3

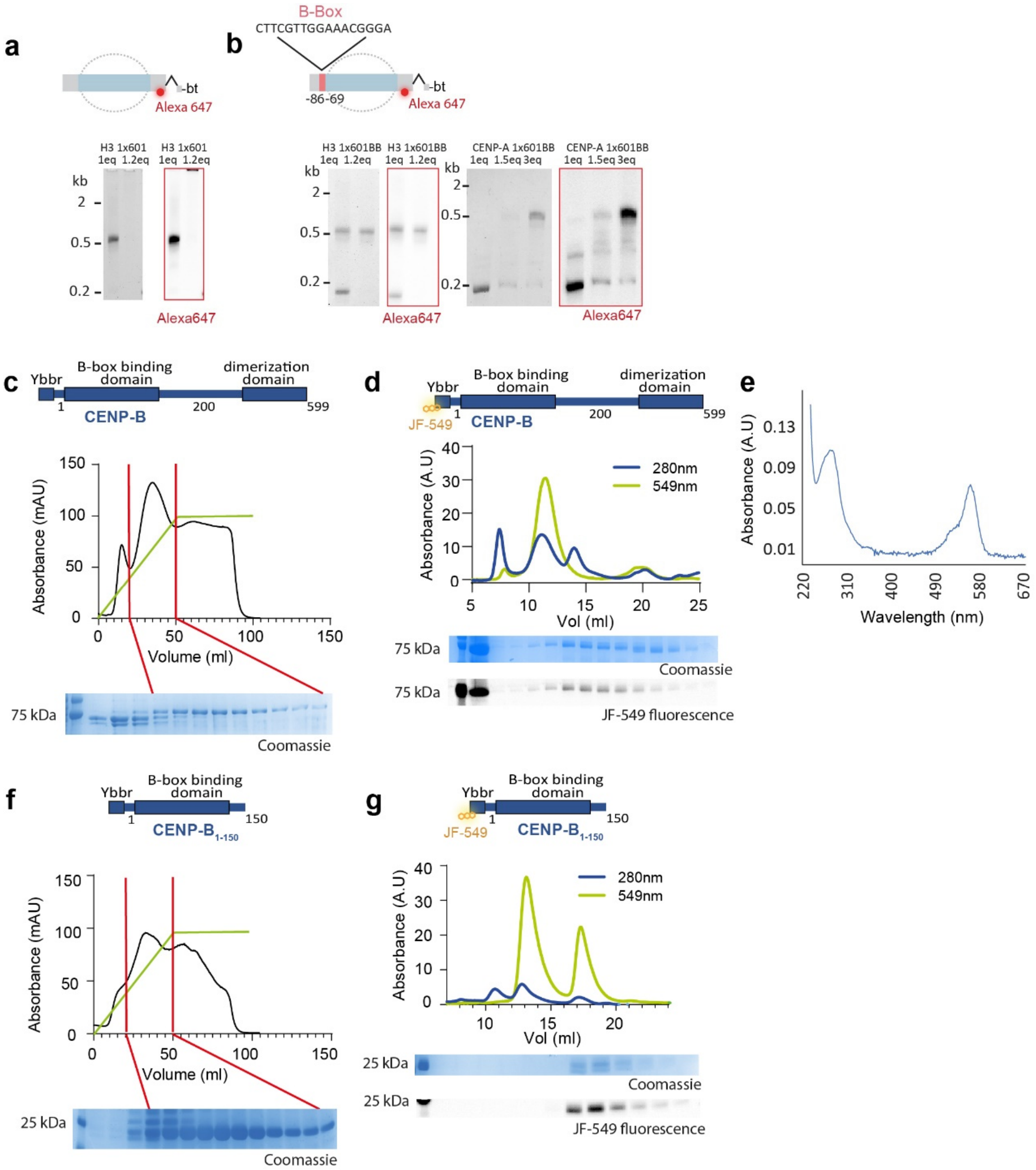

Supplementary Fig. 3 - Mononucleosome formation and CENP-B production. a Scheme and native PAGE analysis of H3 mononucleosome assembly on DNA lacking a B-Box. For the sequence and labeling see Supplementary Tables 1-3. **b** Scheme and native PAGE analysis of H3 and CENP-A mononucleosome assembly on DNA with a positioned B-Box. For the sequence and labeling see Supplementary Tables **1-3**. **c** Gradient Ni-NTA affinity purification of CENP-B (left) and CENP-B 1-150 (right). **d** Purification of labeled of CENP-B. Gel filtration profile shows excitation spectra at 280 nm and emission spectra at 549 nm. Gels show Coomassie (top) and 549nm emission (bottom) lane 1 as input and lane 2-13 as elute of peaks 1, 2, &3 each being void, CENP-B, and sfp synthase. **e** UV-Vis spectrum of labeled CENP-B, enabling a quantification of labeling efficiency (50%). **f** Gradient Ni-NTA affinity purification of CENP-B(1-15). **g** Purification and labelling of JF549-CENP-B(1-150). Gel filtration profile shows excitation spectra at 280nm and emission spectra at 549nm. Gels show Coomassie (top) and 549nm emission (bottom) lane 1 as input and lane 2-13 as elute of peaks 1, 2, &3 each being sfp synthase, CENP-B 1-150, and free dye.

Supplementary Fig. 4

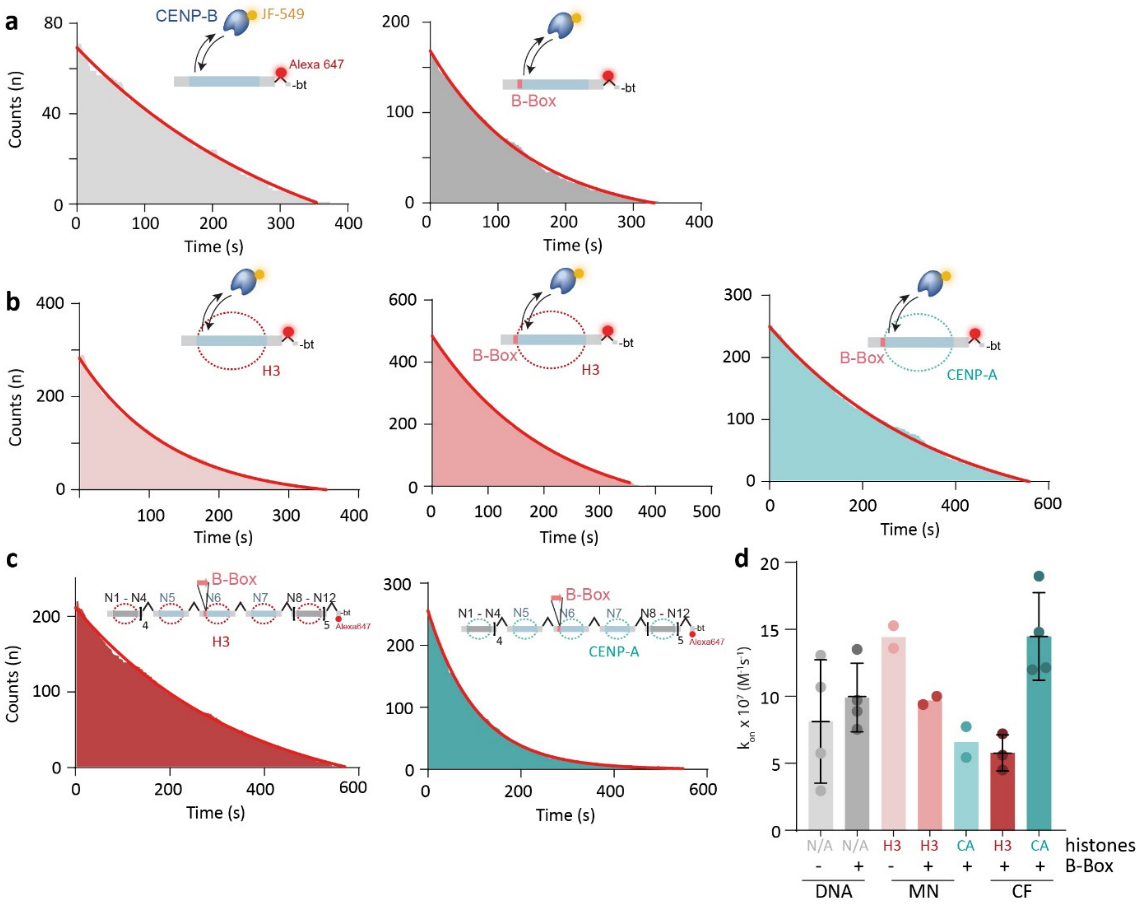

Supplementary Fig. 4 - Binding kinetics of CENP-B. A) Cumulative histogram of dark times (t_{dark}), yielding binding kinetics, and mono-exponential fits of CENP-B binding to 1x601 and 1x601 BB DNA. **b** Example of cumulative histogram of dark times and mono-exponential fits of CENP-B binding to H3 1x601, 1x601 BB mononucleosomes, and CENP-A 1x601BB mononucleosomes. **c** Example of cumulative histogram of dark times and mono-exponential fits of CENP-B binding to H3 or CENP-A containing 12x601 DA3BB chromatin arrays. **d** Specific association kinetics of CENP-B to indicated DNA, nucleosomes (MN) or chromatin fibers (CF). n=4 (DNA), n=2 (MN H3 or CA), n=3 (CF CA), n=4 (CF, H3).

Supplementary Fig. 5

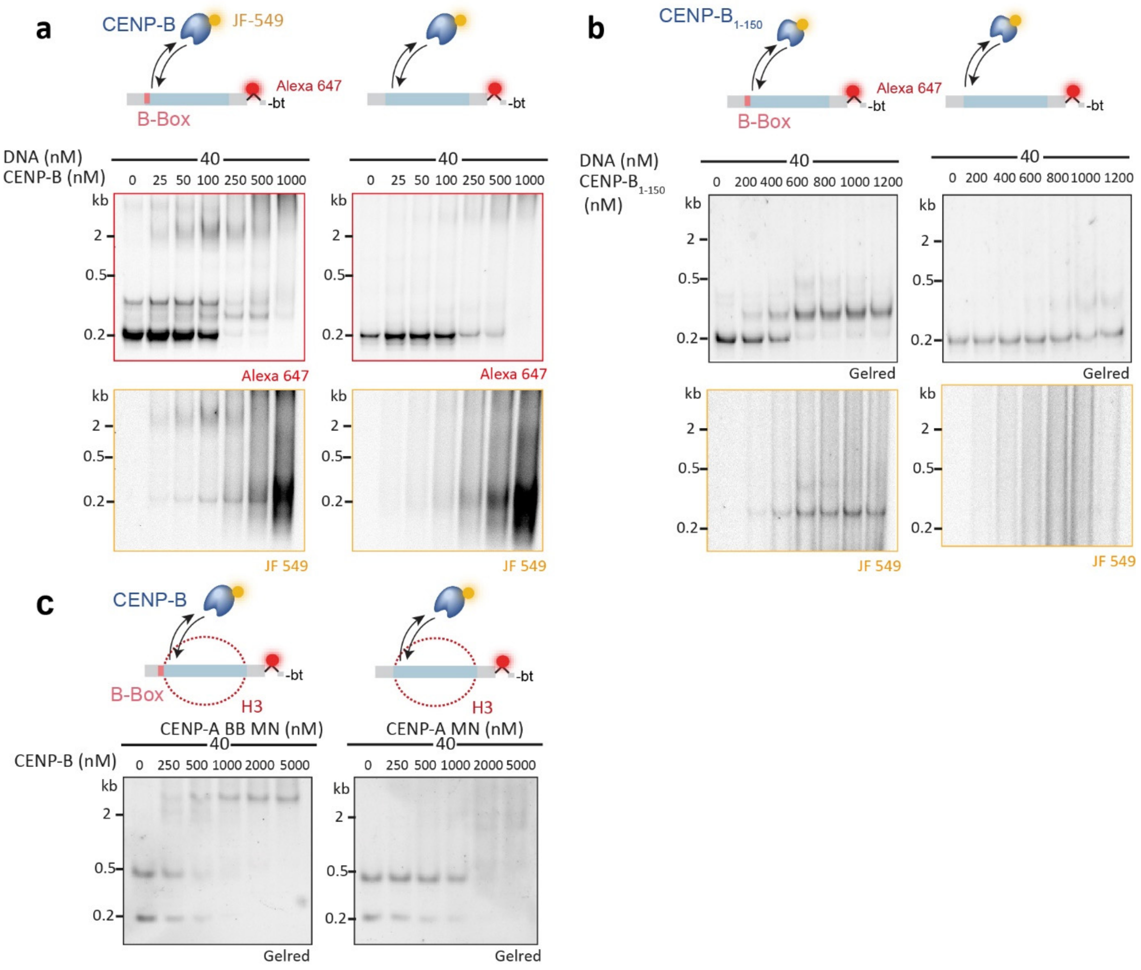

Supplementary Fig. 5 – CENP-B DNA and nucleosome binding. A) CENP-B binding to B-Box (left) and non B-Box (right) containing 1x601 DNA. CENP-B is titrated from 0-1 µM. **b** CENP-B 1-150 binding to B-Box (left) and non B-Box (right) containing 1x601 DNA. CENP-B is titrated from 0-1.2 µM. **c** CENP-B binding to B-Box (left) and non B-Box (right) containing 1x601 CENP-A mononucleosomes. CENP-B is titrated from 0-5 µM.

Supplementary Fig. 6

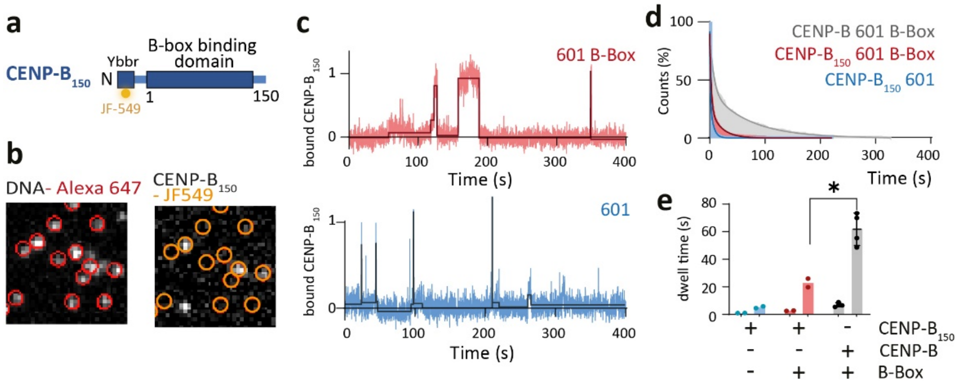

Supplementary Fig. 6: CENP-B kinetochore binding and dimerization domain enhance binding to B-Box DNA. **A**) Domain organization of CENP-B_{150} **b** Representative smTIRF image showing immobilized 601-B-Box DNA in the far-red channel (left, red circles) and CENP-B binding events in the green-orange channel at the same positions (right, orange circles). **c** Representative fluorescence time trace of CENP-B 1-150 binding events to 601-B-Box (top) and non-B-Box DNA (bottom). The traces are fitted and t_{dark} and t_{bright} are determined. **d** Cumulative histogram of CENP-B 1-150 binding to B-Box (red) and non-B-Box DNA (blue), and CENP-B Fl binding to 601 B-Box DNA (grey) fitted by a bi-exponential function (solid line). **e** Specific disassociation time constants ($t_{off,i}$) of CENP-B 1-150 to non-B-Box (blue) and B-Box (red) 601 DNA, and CENP-B Fl to B-Box 601 DNA. n=2 (CENP-B_{150}, - B-Box), n=2 (CENP-B_{150}, + B-Box), n=4 (CENP-B, +B-Box); error bars show mean +/- S.D. *<0.05 using 2-tailed unpaired t-test.

Supplementary Fig. 7

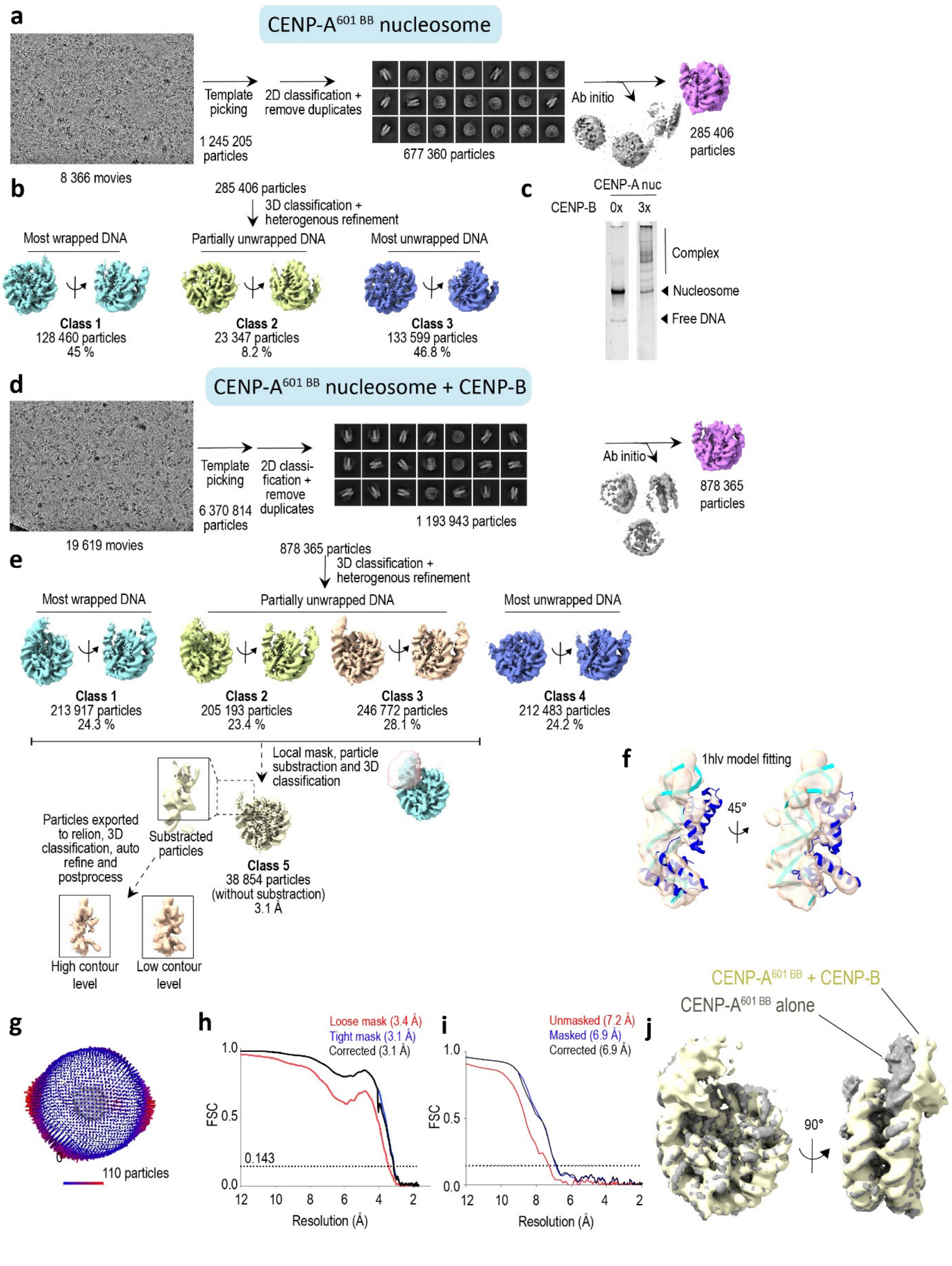

Supplementary Fig. 7: Cryo-EM analysis of CENP-A$^{601\ BB}$ nucleosome in complex with CENP-B. a From left to right: Representative cryo-EM micrograph, subset of selected 2D class averages and the reference map for CENP-A$^{601\ BB}$ nucleosome alone generated using *ab initio* reconstruction. Discarded classes from *ab initio* reconstruction are shown in grey. **b** Flowchart of 3D classification analysis for CENP-A$^{601\ BB}$ nucleosome. After homogeneous refinement of the selected particles from *ab initio* reconstruction, 3D classification followed by heterogeneous refinement was performed to classify the differences at the DNA entry/exit site. Classes are grouped according to DNA conformation at the entry/exit sites. **c** Native gel page showing CENP-A$^{601\ BB}$ nucleosome alone and in complex with CENP-B. The molar ratio of CENP-B/nucleosome is indicated above the lane. **d** From left to right: Representative cryo-EM micrograph, subset of selected 2D class averages and the reference map for CENP-A$^{601\ BB}$ nucleosome in complex with CENP-B generated using *ab initio* reconstruction. Discarded classes from *ab initio* reconstruction are shown in grey. **e** Flowchart of the 3D classification analysis for CENP-A$^{601\ BB}$ nucleosome in complex with CENP-B. The particles were classified to highlight differences at the DNA entry/exit site. Classes showing extra density on the linker DNA, where further 3D classified and refined to get a final resolution of 3.1 Å. The DNA linker region was extracted from the particles using a local mask shown in transparent pink. Extracted particles were locally aligned in cryosparc and then exported to Relion V3.12 for further classification and refinement. Final map for this region is shown in bottom left corner at high and low contour level. **f** Coordinates from the crystal structure of CENP-B DNA binding domain (dark blue) in complex with CENP-B box DNA (cyan) (PDB code: 1HLV) fitted in the final map obtained after postprocessing in Relion. **g** Euler angle distribution of particles used in the 3D reconstruction of CENP-A$^{601\ BB}$ nucleosome in complex with CENP-B (Class 5). **h** The FSC curve of the 3D reconstruction of CENP-A$^{601\ BB}$ nucleosome in complex with CENP-B (Class 5). **i** The FSC curve of the 3D reconstruction of CENP-B/DNA extracted map shown in **f**. **j** Overlay of 3D cryo-EM maps from class 3 in grey (unbound CENP-B) and class 5 in yellow (bound CENP-B) showing different DNA conformation in the presence of CENP-B. For all cryoEM data collection and map refinement statistics, see Supplementary Tables 6-8.

Supplementary Fig. 8

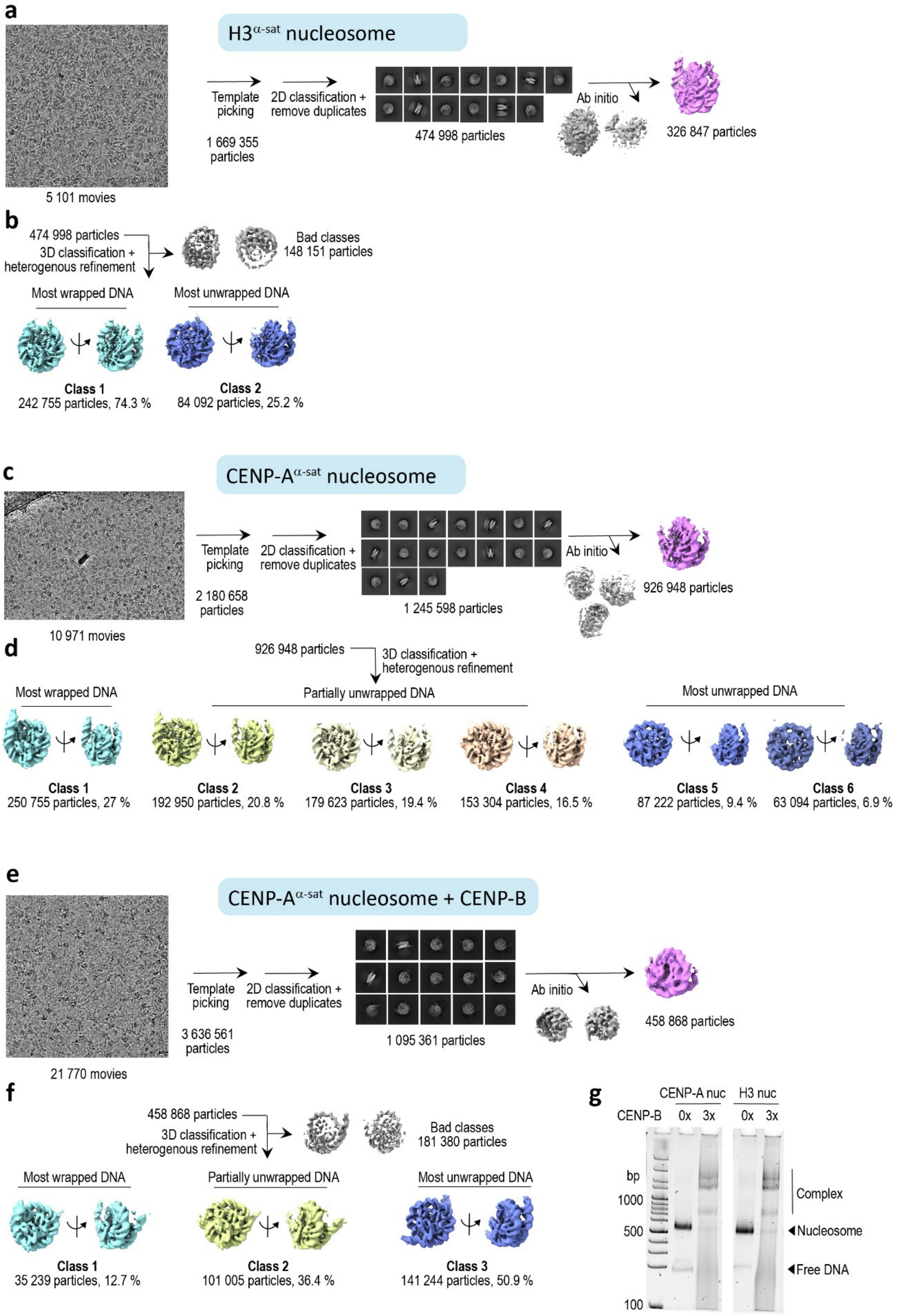

Supplementary Fig. 8: Cryo-EM analysis of H3$^{\alpha\text{-sat}}$ nucleosomes, CENP-A$^{\alpha\text{-sat}}$ nucleosomes in isolation and in complex with CENP-B. a From left to right: Representative cryo-EM micrograph, subset of selected 2D class averages and the reference map for H3$^{\alpha\text{-sat}}$ nucleosome alone generated using *ab initio* reconstruction. Discarded classes from *ab initio* reconstruction are shown in grey. **b** Flowchart of 3D classification analysis for H3$^{\alpha\text{-sat}}$ nucleosome. After homogeneous refinement of the selected particles from *ab initio* reconstruction, 3D classification followed by heterogeneous refinement was performed to classify the differences at the DNA entry/exit site. Classes are grouped according to DNA conformation at the entry/exit sites. 3D classes discarded in the 3D classification process are shown in grey. **c** From left to right: Representative cryo-EM micrograph, subset of selected 2D class averages and the reference map for CENP-A$^{\alpha\text{-sat}}$ nucleosome generated using *ab initio* reconstruction. Discarded classes from *ab initio* reconstruction are shown in grey. **d** Flowchart of the 3D classification analysis for CENP-A$^{\alpha\text{-sat}}$ nucleosome. As in **b**, the particles were classified to highlight differences at the DNA entry/exit site. **e** From left to right: Representative cryo-EM micrograph, subset of selected 2D class averages and the reference map for CENP-A$^{\alpha\text{-sat}}$ nucleosome in complex with CENP-B generated using *ab initio* reconstruction. Discarded classes from *ab initio* reconstruction are shown in grey. **f** Flowchart of the 3D classification analysis for CENP-A$^{\alpha\text{-sat}}$ nucleosome in complex with full-length CENP-B. The particles were classified to highlight differences at the DNA entry/exit site. 3D classes discarded in the 3D classification process are shown in grey. **g** Native PAGE gel showing CENP-A$^{\alpha\text{-sat}}$ and H3$^{\alpha\text{-sat}}$ nucleosome alone and in complex with CENP-B. The molar ratio of CENP-B/nucleosome is shown above each lane. For all cryoEM data collection and map refinement statistics, see Supplementary Tables 6-8.

Supplementary Fig. 9

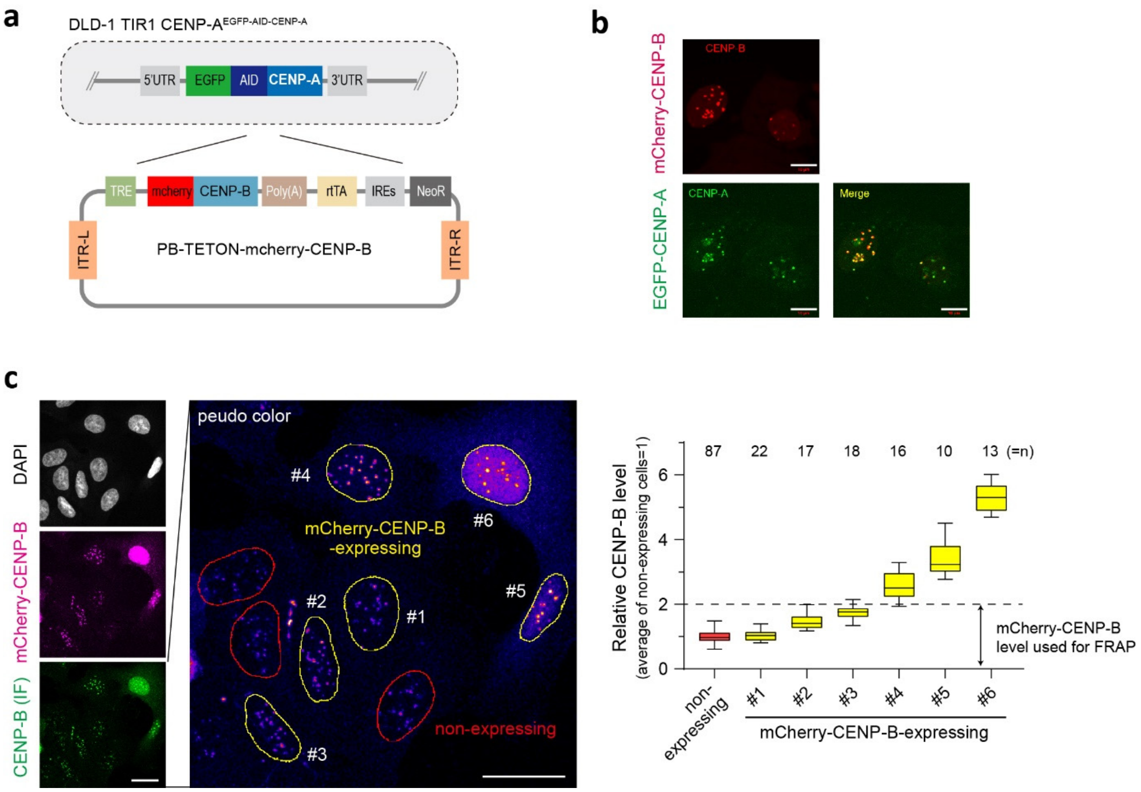

Supplementary Fig. 9 – Establishment of DLD-1 TIR1 CENP-A$^{\text{EGFP-AID-CENP-A}}$ mCherry-CENP-B cell line and expression optimization. a Modification of DLD-1 TIR1 CENP-A$^{\text{EGFP-AID-CENP-A}}$ cell line[5] via the shown PiggyBac vector to express mCherry-CENP-B under a TetOn promoter. **b** Colocalization of mCherry-CENP-B with EGFP-CENP-A. **c** Calibration of CENP-B expression leveld. Left and middle: mCherry-CENP-B (magenta) was expressed by incubation with 50 ng/ml doxycycline for 12 hours. The cells were immunostained with anti-CENP-B antibody (CENP-B(IF); green). The cells expressing or not expressing mCherry-CENP-B were surrounded by yellow and red, respectively. Right: Quantification of CENP-B level at centromeres in mCherry-CENP-B-expressing (yellow) or non-expressing (red) cells is shown. n represents the number of quantified centromeres. For non-expressing cells, n is the total centromere number from five different cells. Box: 25th-75th percentile, line: median, whiskers: Tukey, Cell selection: For FRAP analysis, we only used cells in which the total CENP-B level (mCherry-CENP-B + endogenous CENP-B) was less than two-fold increase compared to cells that do not express mCherry-CENP-B. Scale bars: 20 µm (levels #1-#3, as judged by mCherry emission using pre-determined imaging parameters).

Supplementary Fig. 10

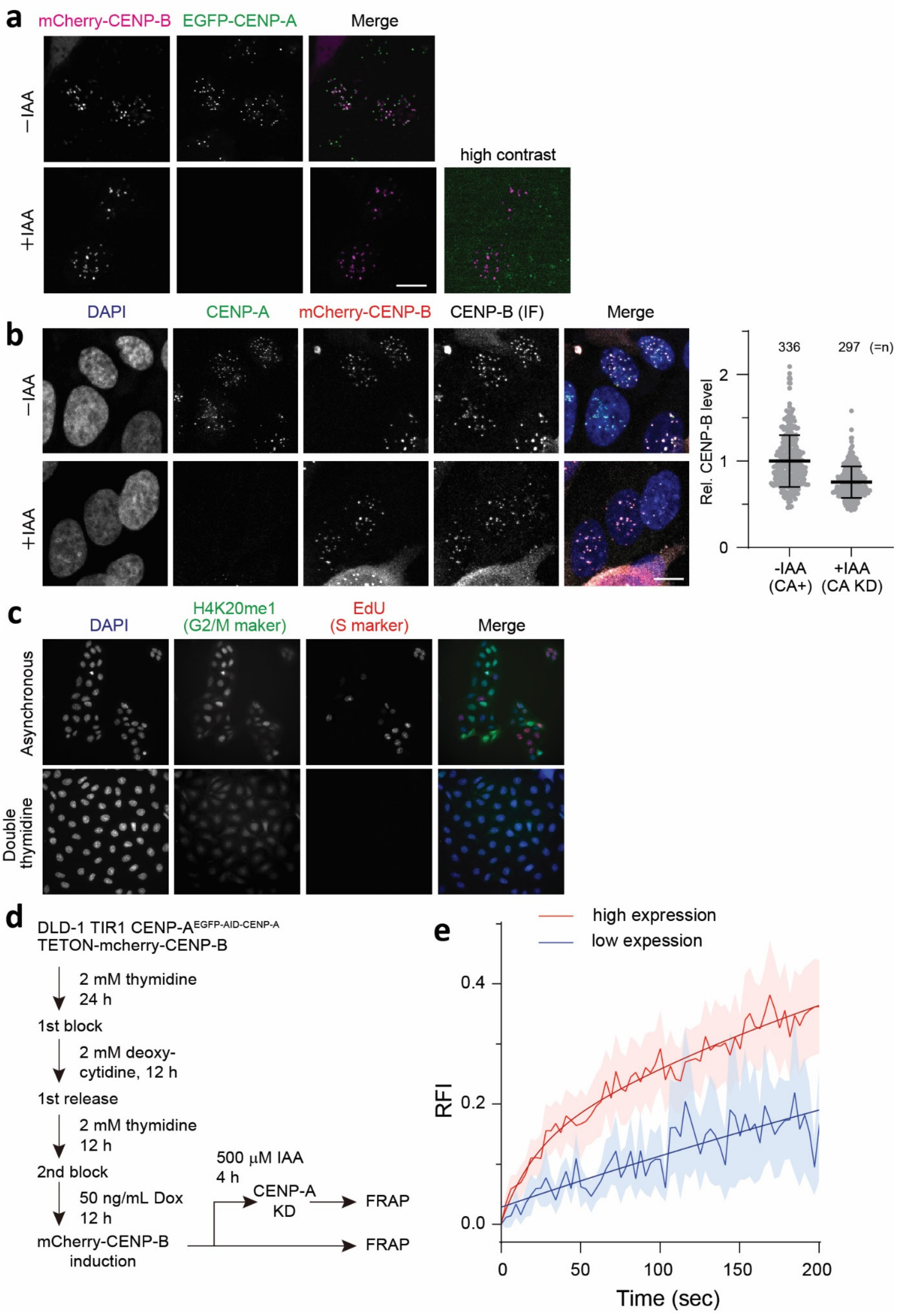

Supplementary Fig. 10 – FRAP experimental conditions.

a CENP-A knockdown by AID system: The cells were incubated for 4 hours in the presence (+IAA) or absence (-IAA) of 500 µM IAA, and observed by confocal microscopy. High contrast image on +IAA condition is shown on the right. CENP-A levels were below the detection limit by microscopy. Scale bar for all images: 10 µm. **B**) Centromeric CENP-B levels are sightly decreased after CENP-A knockdown. The cells were incubated for 4 hours in the presence (+IAA) or absence (-IAA) of 500 µM IAA, stained with anti-CENP-B antibody, and then observed by confocal microscopy. The right graph shows the quantification result of CENP-B levels. The mean value of CENP-B intensity in CA+ cells are set as 1.0. n represents the number of centromere quantified. Scale bar: 10 µm. **c** G1-phase arrest: The cells were arrested in the G1-phase by a double thymidine block. After labeling S-phase cells by EdU, the cells were immuno-stained with an anti-H4K20me1 antibody as a G2/M-phase marker. Scale bar: 50 µm. The absence of EdU staining and H4K20me1 signal in the treated cells (bottom row) demonstrates a successful G1 arrest. **d** Scheme of the experiment, indicating the sequence of inducing CENP-B expression, establishing the dithymidine block for G1 arrest, followed by AID-induced CENP-A depletion and FRAP experiment. **e** Normalized recovery curves for quantitative FRAP measurements of CENP-B at high expression level (red, #6) or low expression level (blue, #1-3). Thick lines indicate mean values and the colored areas indicate the standard error. For high and low expressing cells, 9 and 8 centromeres were used for analysis, respectively. The data for high expressing cells were fit using a dual exponential function (dark red, $\tau_1 = 20 \pm 11$ s, $\tau_2 = 377 \pm 583$ s). The dark blue line indicates a single exponential decay with τ fixed at the fit value from **Fig. 7B**.

Uncropped gel images

Supplemental Fig. 1a

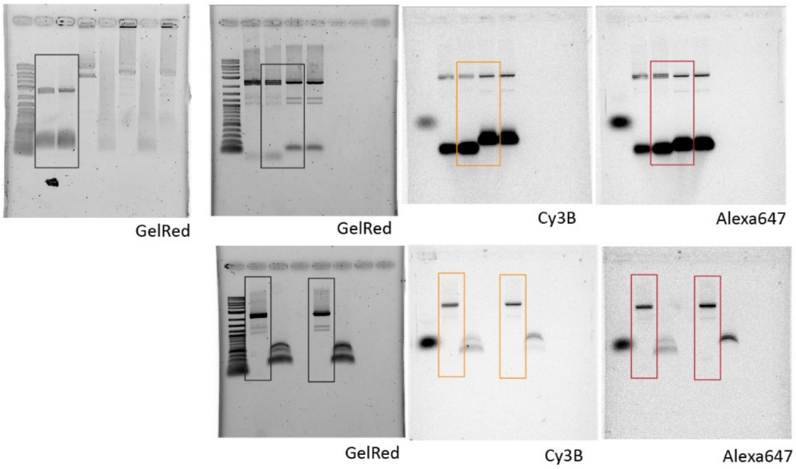

Supplemental Fig. 1b - upper panels & 1c upper panel

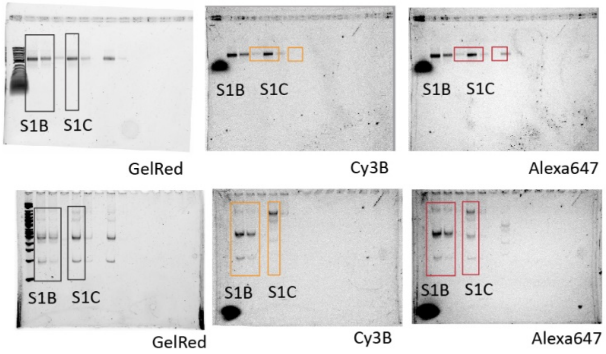

Supplemental Fig. 1b - lower panels

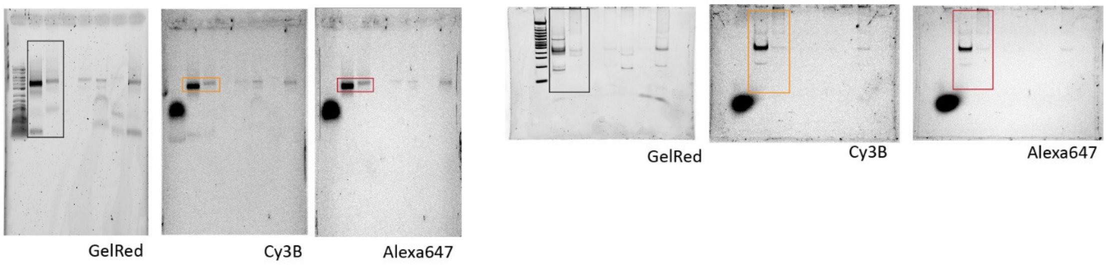

Supplemental Fig. 1c - lower panels

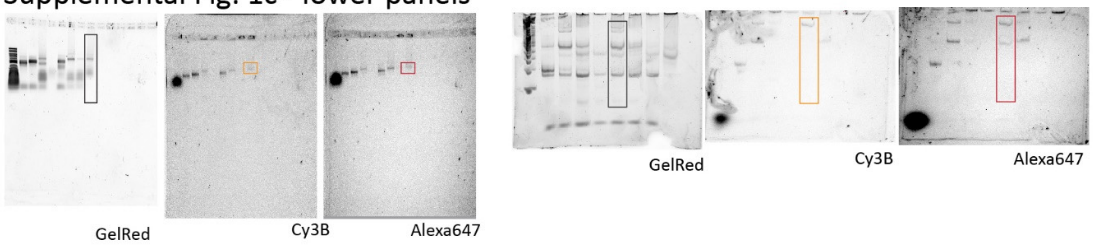

Supplemental Fig. 3a

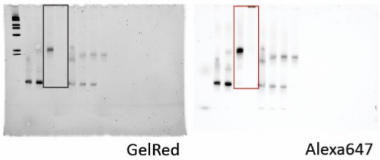

Supplemental Fig. 3b

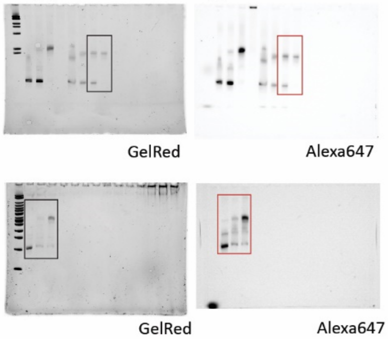

Supplemental Fig. 3c

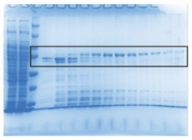

Supplemental Fig. 3d

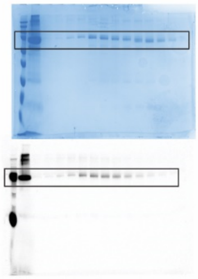

Supplemental Fig. 3f

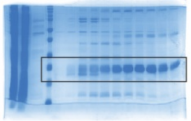

Supplemental Fig. 3g

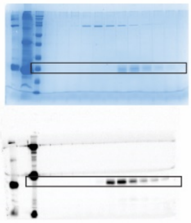

Supplemental Fig. 5a - left panels

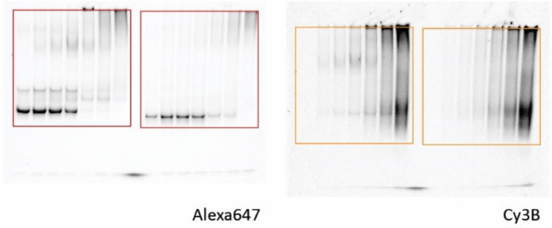

Supplemental 5a - right panels

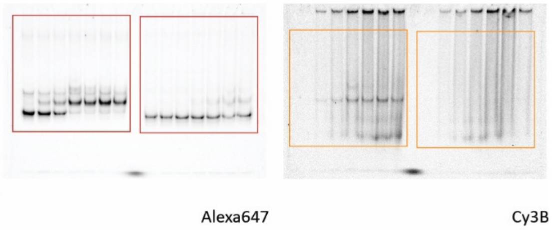

Supplemental 5c

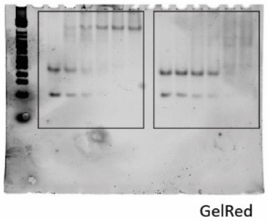

Supplemental Fig. 7c

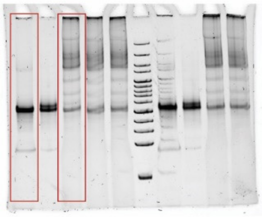

Supplemental Fig. 8g

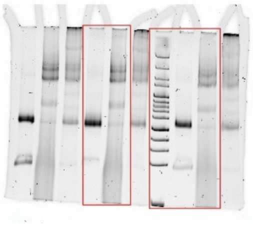

Supplementary references

1. Mivelaz M*, et al.* Chromatin Fiber Invasion and Nucleosome Displacement by the Rap1 Transcription Factor. *Mol Cell* **77**, 488-500 e489 (2020).

2. Banerjee DR*, et al.* Plug-and-Play Approach for Preparing Chromatin Containing Site-Specific DNA Modifications: The Influence of Chromatin Structure on Base Excision Repair. *J Am Chem Soc* **140**, 8260-8267 (2018).

3. Takizawa Y*, et al.* Cryo-EM Structures of Centromeric Tri-nucleosomes Containing a Central CENP-A Nucleosome. *Structure* **28**, 44-53 e44 (2020).

4. Kalinin S*, et al.* A toolkit and benchmark study for FRET-restrained high-precision structural modeling. *Nat Methods* **9**, 1218-1225 (2012).

5. Barra V*, et al.* Phosphorylation of CENP-A on serine 7 does not control centromere function. *Nat Commun* **10**, 175 (2019).
