# Supplementary material for: CENP-A and CENP-B collaborate to create an open centromeric chromatin state
Source: Nat Commun. 2023 Dec 12;14:8227. doi: 10.1038/s41467-023-43739-5 (PMC10716449; doi:10.1038/s41467-023-43739-5)
Supplement: Supplementary file 3 — Description of Additional Supplementary Files [file 41467_2023_43739_MOESM3_ESM.pdf]

Description of Additional Supplementary files

Supplementary Movie 1: CENP-B binding destabilizes the conformation of 601 B-Box linker DNA. Alignment of different cryo-EM 3D classes (classes 1,2,3 and 5 from figure S7) of CENP-A^{601BB}-CENP-B complex showing different adopted conformations of the linker DNA upon CENP-B binding.
