# Supplementary material for: CENP-A and CENP-B collaborate to create an open centromeric chromatin state
Source: Nat Commun. 2023 Dec 12;14:8227. doi: 10.1038/s41467-023-43739-5 (PMC10716449; doi:10.1038/s41467-023-43739-5)
Supplement: Supplementary file 5 — Reporting Summary [file 41467_2023_43739_MOESM5_ESM.pdf]

nature portfolio

Corresponding author(s): Nikolina Sekulic, Beat Fierz

Last updated by author(s): 10/24/2023

Reporting Summary

Nature Portfolio wishes to improve the reproducibility of the work that we publish. This form provides structure for consistency and transparency in reporting. For further information on Nature Portfolio policies, see our Editorial Policies and the Editorial Policy Checklist.

Statistics

For all statistical analyses, confirm that the following items are present in the figure legend, table legend, main text, or Methods section.

n/a	Confirmed	
☐	☒	The exact sample size (*n*) for each experimental group/condition, given as a discrete number and unit of measurement
☐	☒	A statement on whether measurements were taken from distinct samples or whether the same sample was measured repeatedly
☐	☒	The statistical test(s) used AND whether they are one- or two-sided<br>*Only common tests should be described solely by name; describe more complex techniques in the Methods section.*
☒	☐	A description of all covariates tested
☒	☐	A description of any assumptions or corrections, such as tests of normality and adjustment for multiple comparisons
☐	☒	A full description of the statistical parameters including central tendency (e.g. means) or other basic estimates (e.g. regression coefficient) AND variation (e.g. standard deviation) or associated estimates of uncertainty (e.g. confidence intervals)
☐	☒	For null hypothesis testing, the test statistic (e.g. *F*, *t*, *r*) with confidence intervals, effect sizes, degrees of freedom and *P* value noted<br>*Give P values as exact values whenever suitable.*
☒	☐	For Bayesian analysis, information on the choice of priors and Markov chain Monte Carlo settings
☒	☐	For hierarchical and complex designs, identification of the appropriate level for tests and full reporting of outcomes
☒	☐	Estimates of effect sizes (e.g. Cohen's *d*, Pearson's *r*), indicating how they were calculated

Our web collection on statistics for biologists contains articles on many of the points above.

Software and code

Policy information about availability of computer code

Data collection	NIS-elements 5.21.03 64-bit (Nikon) and Solis 4.27.30007.0(Andor) were used for microscopy data acquisition, Labview 14.0.1 32-bit (National Instruments) was used for microscope control, cyoEM data were acquired using EPU v2.24.0 or v3.2.0 (Thermo Fisher Scientific), FRAP data was recorded using Zen 2.3 SP1 (Zeiss)
Data analysis	Single molecule data were analyzed using MATLAB 2018b (Mathworks), kinetic data was analyzed using Origin 2020b and GraphPad Prism 10 (Dotmatics), cryoEM datasets were analyzed using CryoSPARC 4.1.2 and Relion v3.12, microscopy data was further analyzed using Fiji v.1.54f

For manuscripts utilizing custom algorithms or software that are central to the research but not yet described in published literature, software must be made available to editors and reviewers. We strongly encourage code deposition in a community repository (e.g. GitHub). See the Nature Portfolio guidelines for submitting code & software for further information.

Data

Policy information about availability of data

All manuscripts must include a data availability statement. This statement should provide the following information, where applicable:

- Accession codes, unique identifiers, or web links for publicly available datasets
- A description of any restrictions on data availability
- For clinical datasets or third party data, please ensure that the statement adheres to our policy

The single molecule data generated in this study have been deposited in zenodo.org under the DOI http://doi.org/10.5281/zenodo.8233663, http://

doi.org/10.5281/zenodo.8233659, http://doi.org/10.5281/zenodo.8233667, http://doi.org/10.5281/zenodo.8233665, http://doi.org/10.5281/zenodo.8239003, http://doi.org/10.5281/zenodo.8250004, http://doi.org/10.5281/zenodo.8239482, http://doi.org/10.5281/zenodo.8239639, http://doi.org/10.5281/zenodo.8239637, http://doi.org/10.5281/zenodo.8239613. Source data for Figures 1-7 and Supplementary Figures S4, S6 and S9, are provided with the paper as a Supplementary File. CryoEM maps were deposited to EMDB under accession no. XXXX (http://doi.org/YYYY)

Research involving human participants, their data, or biological material

Policy information about studies with human participants or human data. See also policy information about sex, gender (identity/presentation), and sexual orientation and race, ethnicity and racism.

Reporting on sex and gender	N/A
Reporting on race, ethnicity, or other socially relevant groupings	N/A
Population characteristics	N/A
Recruitment	N/A
Ethics oversight	N/A

Note that full information on the approval of the study protocol must also be provided in the manuscript.

Field-specific reporting

Please select the one below that is the best fit for your research. If you are not sure, read the appropriate sections before making your selection.

☒ Life sciences ☐ Behavioural & social sciences ☐ Ecological, evolutionary & environmental sciences

For a reference copy of the document with all sections, see nature.com/documents/nr-reporting-summary-flat.pdf

Life sciences study design

All studies must disclose on these points even when the disclosure is negative.

Sample size	no sample size calculation was performed. All experiments have been independently replicated, and the number of replicates is indicated in the figure legends.
Data exclusions	single-molecule data was excluded/selected based on clear criteria, described in detail in the supplementary materials; 'FRET data analysis'
Replication	all data was replicated, the number of replicates is provided in the figure legends
Randomization	Randomization was not relevant to this study because experiments were not performed on particular populations and all experiment were independently repeated-
Blinding	data was not analyzed by blinded investigators, as data analysis criteria were predefined and data analysis was quantitative (numerical measurements). Thus subjective bias is not an issue.

Reporting for specific materials, systems and methods

We require information from authors about some types of materials, experimental systems and methods used in many studies. Here, indicate whether each material, system or method listed is relevant to your study. If you are not sure if a list item applies to your research, read the appropriate section before selecting a response.

Materials & experimental systems

n/a	Involved in the study	
☐	☒	Antibodies
☐	☒	Eukaryotic cell lines
☒	☐	Palaeontology and archaeology
☒	☐	Animals and other organisms
☒	☐	Clinical data
☒	☐	Dual use research of concern
☒	☐	Plants

Methods

n/a	Involved in the study	
☒	☐	ChIP-seq
☒	☐	Flow cytometry
☒	☐	MRI-based neuroimaging

Antibodies

Antibodies used	anti-H4K20me1 (clone ID: 22G3, a kind gift from Dr. Hiroshi Kimura, Tokyo Tech University), anti-CENP-B (clone ID: 5e6c1, a kind gift from Dr. Hiroshi Masumoto, Kazusa DNA Research Institute)
Validation	anti-H4K20me1: Sato, Y., Kujirai, T., Arai, R., Asakawa, H., Ohtsuki, C., Horikoshi, N., Yamagata, K., Ueda, J., Nagase, T., Haraguchi, T., Hiraoka, Y., Kimura, A., Kurumizaka, H. & Kimura, H. A Genetically Encoded Probe for Live-Cell Imaging of H4K20 Monomethylation. J Mol Biol 428, 3885-3902 (2016).<br>anti-CENP-B: Otake, K., Ohzeki, J.I., Shono, N., Kugou, K., Okazaki, K., Nagase, T., Yamakawa, H., Kouprina, N., Larionov, V., Kimura, H., Earnshaw, W.C. & Masumoto, H. CENP-B creates alternative epigenetic chromatin states permissive for CENP-A or heterochromatin assembly. J Cell Sci 133(2020).

Eukaryotic cell lines

Policy information about cell lines and Sex and Gender in Research

Cell line source(s)	DLD-1 TIR1 CENP-AEGFP-AID-CENP-A: A kind gift of Prof. Daniele Fachinetti, Institute Curie
Authentication	the cell line was used without authentication
Mycoplasma contamination	the cell lines were monthly checked for mycoplasma contamination, which was always negative
Commonly misidentified lines (See ICLAC register)	no commonly misidentified cell lines were used
